# Origin of wheat B-genome chromosomes inferred from RNA sequencing analysis of leaf transcripts from section Sitopsis species of *Aegilops*

**DOI:** 10.1093/dnares/dsy047

**Published:** 2019-01-30

**Authors:** Yuka Miki, Kentaro Yoshida, Nobuyuki Mizuno, Shuhei Nasuda, Kazuhiro Sato, Shigeo Takumi

**Affiliations:** 1 Graduate School of Agricultural Science, Kobe University, Kobe, Japan; 2 Graduate School of Agriculture, Kyoto University, Kyoto, Japan; 3 Institute of Plant Science and Resources, Okayama University, Kurashiki, Japan

**Keywords:** chromosomal synteny, genome-wide polymorphisms, genome differentiation, RNA sequencing, wheat

## Abstract

Dramatic changes occasionally occur in intergenic regions leading to genomic alterations during speciation and will consequently obscure the ancestral species that have contributed to the formation of allopolyploid organisms. The S genome of five species of section Sitopsis of genus *Aegilops* is considered to be an origin of B-genome in cultivated tetraploid and hexaploid wheat species, although its actual donor is still unclear. Here, we attempted to elucidate phylogenetic relationship among Sitopsis species by performing RNA sequencing of the coding regions of each chromosome. Thus, genome-wide polymorphisms were extensively analyzed in 19 accessions of the Sitopsis species in reference to the tetraploid and hexaploid wheat B genome sequences and consequently were efficiently anchored to the B-genome chromosomes. The results of our genome-wide exon sequencing and resultant phylogenetic analysis indicate that *Ae. speltoides* is likely to be the direct donor of all chromosomes of the wheat B genome. Our results also indicate that the genome differentiation during wheat allopolyploidization from S to B proceeds at different speeds over the chromosomes rather than at constant rate and recombination could be a factor determining the speed. This observation is potentially generalized to genome differentiation during plant allopolyploid evolution.

## 1. Introduction

Common wheat (*Triticum aestivum* L., genome constitution AABBDD), a major food crop, is an allohexaploid species derived via allopolyploid speciation through interspecific crossing between cultivated tetraploid wheat *Triticum turgidum* L. (AABB) and its diploid relative, *Aegilops tauschii* Coss. (DD).^1–4^ The cultivated tetraploid form was domesticated from the wild tetraploid wheat *T. turgidum* subspecies *dicoccoides* (AABB), which was thought to be derived through interspecific hybridization between wild diploid progenitors of the A and B genomes. The A genome donor was the wild diploid wheat *T. urartu*,^5^^,^^6^ and the B genome could have been contributed by *Ae. speltoides* Tausch (SS).^7–10^ However, the origin of the B genome remains unclear, despite extensive research over the past few decades. The cytoplasmic genomes of allopolyploid wheat species were almost certainly transmitted from *Ae. speltoides*,^11^^,^^12^ indicating that at least *Ae. speltoides* contributed to establishment of the nuclear genome of allopolyploid wheat.

The indefinite origin of the wheat B genome is due to failure of homoeologous chromosome pairing between the B genome of allopolyploid wheat and the S genome of *Ae. speltoides* during meiosis in the respective interspecific hybrids.^13^^,^^14^ In addition, the section Sitopsis of *Aegilops* includes four wild diploid species, *Ae. bicornis* Jaub. et Spach. (S^b^S^b^), *Ae. searsii* Feldman et Kislev ex Hammer (S^s^S^s^), *Ae. sharonensis* Eig (S^l^S^l^), and *Ae. longissima* Schweinf. & Muschl. (S^l^S^l^), except *Ae. speltoides*. Of the five Sitopsis species that share the S genome,^15^ only *Ae. speltoides* of the subsection Truncata is cross-pollinating, whereas the other four subsection Emarginata species are self-pollinating. Two subspecies of *Ae. speltoides* (*ligustica* and *speltoide*s; syn. *Ae. aucheri* Boiss.) have been defined to date,^16^^,^^17^ and they can be distinguished at least in part by a single locus, *Lig*, on chromosome 3S, which controls spike morphology.^18^ The two Emarginata species, *Ae. longissima* and *Ae. sharonensis*, are quite closely related and recognized as forming one complex.^19–22^ The F_1_ hybrid plants among Sitopsis species show incomplete homoeologous pairing during meiosis,^23^ suggesting that differentiation to the modified S genome occurred during diversification of the Sitopsis species. Some chromosomal rearrangements in the S genome, including translocations, have been reported in Sitopsis species.^20^^,^^24^ In addition, the B and S genomes can exhibit differences in the pattern of transposable element insertion.^25^^,^^26^ Structural differences in intergenic regions have therefore increased the phylogenetic distance between the B genome of polyploid wheat and the S genome of *Ae. speltoides*. Constitutive heterochromatic regions detected by chromosome staining are much more abundant in B-genome chromosomes than those of the A and D genomes,^27^^,^^28^ and the staining patterns of B-genome chromosomes appear to differ from those of S-genome chromosomes of *Ae. speltoides*.^27^ The difference in heterochromatin bands results in reduced pairing between B and S homoeologous chromosomes.^28^^,^^29^ These structural modifications and distinct heterochromatin distribution have made it difficult to elucidate the origin of the B genome and assess the relationship between the Sitopsis genomes and B genome.

Molecular phylogenetic studies based on nuclear DNA polymorphisms (e.g. restriction fragment length polymorphisms [RFLPs] and amplified fragment length polymorphisms [AFLPs]) have revealed that the two subsections of Sitopsis are extensively differentiated^7^^,^^21^^,^^30^ and that the wheat B genome is much more closely related to the S genome of *Ae. speltoides* than to the other modified S genomes of subsection Emarginata species.^7^^,^^21^ Analyses of nucleotide sequence polymorphisms in single-copy genes also supported the hypothesis that *Ae. speltoides* is the donor of the B genome in allopolyploid wheat.^31^^,^^32^ In contrast, a few reports have suggested a polyphyletic origin of the wheat B genome via the introgression of several parental Sitopsis species.^33–35^ For example, a low copy number, non-coding sequence located in the region comprising 19% of the distal portion of the long arm of chromosome 3B exists only in *Ae. searsii* among all Sitopsis species.^33^ Moreover, nucleotide sequence analyses have revealed increased divergence in the B genome of modern common wheat compared with *Ae. speltoides*, and this divergence is thought to be a result of polyploidization events affecting B-genome evolution.^34^ Thus, the phylogenetic relationship between the B and S genomes of section Sitopsis should be reconsidered based on the polymorphisms of each limited chromosomal region as well as those covering the entire chromosomal regions of the B and S genomes.

RNA sequencing is an effective approach for surveying large number of genome-wide polymorphisms derived only from the exon sequences in *Aegilops* species.^36–39^ In RNA sequencing of *Aegilops* species, polymorphisms identified without any reference genome information can be efficiently anchored to the homoeologous chromosomes of related species, such as common wheat and barley, based on conserved chromosomal synteny.^40^ Here, we conducted RNA sequencing analyses of leaf transcripts from section Sitopsis species to avoid the intergenic and repetitive sequences of wheat chromosomes. The objectives of the present study were to (i) identify genome-wide polymorphisms in the Sitopsis genomes, (ii) elucidate the phylogenetic relationship among Sitopsis species, and (iii) determine the wheat B-genome origin based on genome-wide polymorphisms anchored putatively to each chromosome of the B genome.

## 2. Materials and methods

### 2.1. Plant materials

Three accessions of *Ae. speltoides* ssp. *ligustica* (SS genome), four accessions of *Ae. speltoides* ssp. *speltoides* (SS genome), two accessions of *Ae. bicornis* (S^b^S^b^ genome), three accessions of *Ae. longissima* (S^l^S^l^ genome), three accessions of *Ae. sharonensis* (S^l^S^l^ genome), and four accessions of *Ae. searsii* (S^s^S^s^ genome) were chosen as representatives of each species from the collection of the section Sitopsis at the National Bio Resource Project–Wheat, Japan (Table 1). These accessions of Sitopsis species were originally collected in the Middle East (Supplementary Fig. S1). A tetraploid wheat (*T. turgidum*) cultivar Langdon (AABB genome) was also used in this study. *Triticum urartu* KU-199-5 (AA genome), *Ae. umbellulata* KU-4017 (UU genome), and *Ae. tauschii* KU-2075 (DD genome) were used as outgroup species.

**Table 1:** List of the 19 accessions in the section Sitopsis used in RNA-seq analyses

Species	Accession number	Origins	Mating systems
*Aegilops speltoides* ssp. *speltoides*	KU-2208A	Turkey	Outcrossing
	KU-14601	Israel	Outcrossing
	KU-14605	Israel	Outcrossing
	KU-12963a	Syria	Outcrossing
*Ae. speltoides* ssp. *ligustica*	KU-2236	Turkey	Outcrossing
	KU-7716	Iraq	Outcrossing
	KU-7848	Iraq	Outcrossing
*Ae. bicornis*	KU-5784	Egypt	Self-pollinating
	KU-14613	Israel	Self-pollinating
*Ae. longissima*	KU-5752	Jordan	Self-pollinating
	KU-14624	Israel	Self-pollinating
	KU-14635	Israel	Self-pollinating
*Ae. searsii*	KU-5755	Syria	Self-pollinating
	KU-6142	Jordan	Self-pollinating
	KU-6143	Jordan	Self-pollinating
	KU-14651	Israel	Self-pollinating
*Ae. sharonensis*	KU-14661	Israel	Self-pollinating
	KU-14663	Egypt	Self-pollinating
	KU-14668	Israel	Self-pollinating

### 2.2. RNA sequencing

Total RNA was extracted using Sepasol-RNA I Super G (Nacalai Tesque, Kyoto, Japan) from leaves of 2- to 3-month-old plants grown in a glass house. The extracted RNA was treated with DNase I at 37 °C for 20 min, after which paired-end libraries for RNA sequencing were constructed from 6 to 10 µg of total RNA using a TruSeq RNA Library Preparation kit v2 (Illumina, San Diego, CA, USA) according to a previously reported protocol^41^ and then sequenced with 300-bp paired-end reads on an Illumina MiSeq sequencer. The obtained reads were deposited in the DDBJ Sequence Read Archive under accession number DRA007097. RNA sequencing data for the outgroup species (300-bp paired-end reads) were obtained from the DDBJ Sequence Read Archives: BioProject PRJDB4683 for *Ae. tauschii* KU-2075 and DRA006404 for *Ae. umbellulata* KU-4017.

### 2.3. Quality control, alignment of paired-end reads, de novo transcriptome assembly, and single-nucleotide polymorphism (SNP)/insertion-deletion (indel) calling

FASTQC software (https://www.bioinformatics.babraham.ac.uk/projects/fastqc/ (6 July 2017, date last accessed)) was used to evaluate sequencing quality of the reads from each of the sequenced samples. Adapter sequences, low-quality bases with an average quality score per 4 bp of <30, and reads of less than 100 bp were removed using Trimmomatic software, version 0.33,^42^ and only filtered paired reads were retained for subsequent analyses. The filtered reads were aligned to the reference B genome sequences of *T. aestivum* cv. Chinese Spring^43^ using HISAT2 software, version 2.1.0.^44^ To select uniquely mapped reads for SNP and indel calling, reads with a mapping quality of <40 were filtered out using SAMtools.^45^ SNPs and indels were called using Coval^46^ under the same criterion reported by Nishijima et al.^38^; the depth of read coverage was ≥10, and >95% of the mapped reads designated different nucleotide sequences from the reference sequences. To obtain non-redundant SNPs, we selected the positions of SNPs at which the read depth was ≥10 and there were no ambiguous nucleotides in any of the samples. We prepared two sets of non-redundant SNPs for construction of phylogenetic trees for intra- and interspecific comparisons of nucleotide variations. One was estimated in all of the samples, including A, D, U genome species, and the other was in the section of Sitopsis species and the wheat B genome. The distribution of SNPs/indels was visualized on the physical map of the B genome using CIRCOS^47^ and R statistical software. *De novo* transcriptome assembly of *Ae. speltoides* ssp. *ligustica* KU-7716 was performed using Bridger.^48^ Unmapped reads of *Ae. speltoides* ssp. *ligustica* KU-7716 were aligned to its assembled transcripts using Bowtie.^49^ If the breadth coverage of the unmapped reads over a transcript is 100%, its corresponding annotated transcript was searched in the published transcripts of *T. aestivum* cv. Chinese Spring (iwgsc_refseqv1.0)^43^ using BLASTN.^50^

### 2.4. Construction of phylogenetic trees

Neighbour-joining (NJ) and maximum-likelihood (ML) phylogenetic trees were constructed using Molecular Evolutionary Genetics Analysis (MEGA) software, version 5.05.^51^ A Kimura 2-parameter model was used as the substitution model for tree construction. To assess node reliability in the trees, bootstrap probability was calculated from 1,000 bootstrap replicates. To construct phylogenetic trees for each chromosomal segment, the chromosomal regions of the B genome were divided into ranges of 60 Mbp each, generating a total of 86 segments that included 124–931 non-redundant SNPs and 79–442 informative polymorphic sites (Supplementary Table S1). NJ trees for each subset of non-redundant SNPs were constructed using MEGA software. Phylogenetic network trees were constructed using SplitsTree4.^52^

### 2.5. Nucleotide divergence between Sitopsis species/subspecies

To estimate nucleotide divergence between Sitopsis species/subspecies, two distance parameters were calculated: fixed substitutions between species^53^ and average number of nucleotide differences between species.^54^ To clarify positional changes in nucleotide divergence between Sitopsis species and the B genome of *T. aestivum*, the chromosomal regions of the B genome were divided into ranges of 20 Mbp each. A total of 262 segments of subsets of non-redundant SNPs in Sitopsis species and the B genome of *T. aestivum* cv. Chinese Spring were obtained. The fixed nucleotide differences and average number of nucleotide differences between Sitopsis species and the B genome were estimated for each subset of non-redundant SNPs. Non-synonymous fixed nucleotide differences were estimated using the variant annotation and effect prediction tool SnpEff.^55^ The number of genes per 20 Mbp was counted based on the Chinese Spring annotation.^43^

## 3. Results and discussion

### 3.1. RNA sequencing detected numerous SNPs in Sitopsis species and the common wheat B genome

**Figure 1:**
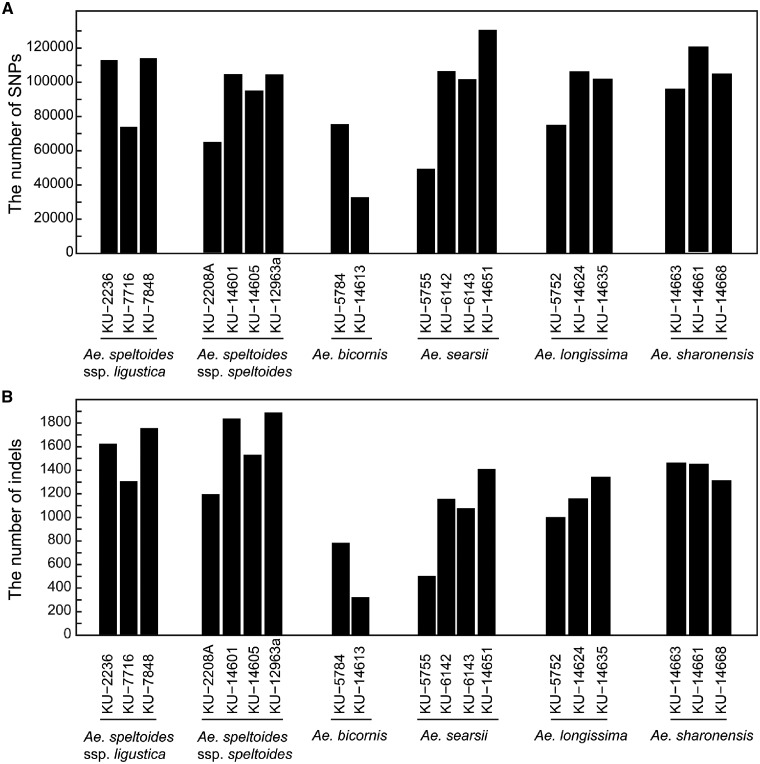
Bar charts illustrating the number of SNPs (A) and indels (B) between the B genome of *T. aestivum* cv. Chinese Spring and each of the 19 accessions of section Sitopsis species.

To identify genome-wide SNPs and indels in Sitopsis species and the wheat B genome, RNA sequencing of 19 representative accessions of the five Sitopsis species was performed, generating 2–3 million filtered paired reads for each species (Table 2). Of these short reads, 67–81% were uniquely aligned to the B genome sequences of Chinese Spring, and 32,836–130,687 SNPs and 323–1,890 indels were identified. The fewest SNPs and indels were found in *Ae. bicornis*, whereas the other four species had similar numbers (Fig. 1). High within-species variance in the number of SNPs and indels was detected but considered a potential artifact because the number of filtered reads differed among the tested accessions (Table 2). To examine this possibility, the correlation between the number of filtered reads and SNPs/indels was determined. However, no correlation was observed between the number of SNPs and indels and the number of filtered reads, suggesting that the high variance is a genetic characteristic of section Sitopsis (Supplementary Fig. S2).

**Table 2:** Summary of RNA sequencing data for the 19 accessions in the section Sitopsis

Species	Accession number	Read pairs	Filtered read pairs	Alignment rate (%)^a^
*Aegilops speltoides* ssp. *speltoides*	KU-2208A	4,317,201	2,721,855 (63.05%)	74.77
	KU-14601	5,279,430	3,088,916 (58.51%)	78.29
	KU-14605	4,005,919	2,480,301 (61.92%)	77.17
	KU-12963a	4,779,168	3,041,573 (63.64%)	70.94
*Ae. speltoides* ssp. *ligustica*	KU-2236	4,378,445	2,313,014 (52.83%)	74.40
	KU-7716	4,036,660	2,553,962 (63.27%)	67.92
	KU-7848	4,962,422	2,843,436 (57.30%)	77.83
*Ae. bicornis*	KU-5784	4,470,520	2, 713,658 (60.70%)	80.71
	KU-14613	4,567,698	2,881,072 (63.07%)	81.04
*Ae. longissima*	KU-5752	4,371,287	2,490,621 (56.98%)	78.48
	KU-14624	5,012,064	2,865,373 (57.17%)	73.05
	KU-14635	4,623,166	2,660,230 (57.54%)	75.52
*Ae. searsii*	KU-5755	5,240,442	3,150,732 (60.12%)	81.23
	KU-6142	5,024,725	3,108,286 (61.86%)	77.75
	KU-6143	4,852,741	2,776,399 (57.21%)	78.16
	KU-14651	5,345,759	3,178,031 (59.45%)	77.87
*Ae. sharonensis*	KU-14661	6,500,270	3,241,926 (49.87%)	75.55
	KU-14663	8,544,460	4,157,728 (48.66%)	78.27
	KU-14668	10,079,695	4,891,456 (48.53%)	75.25

a The aliment rate against the B genome of *T. aesti*vum cv. Chinese Spring was calculated with HISAT2 version 2.1.0.

**Figure 2:**
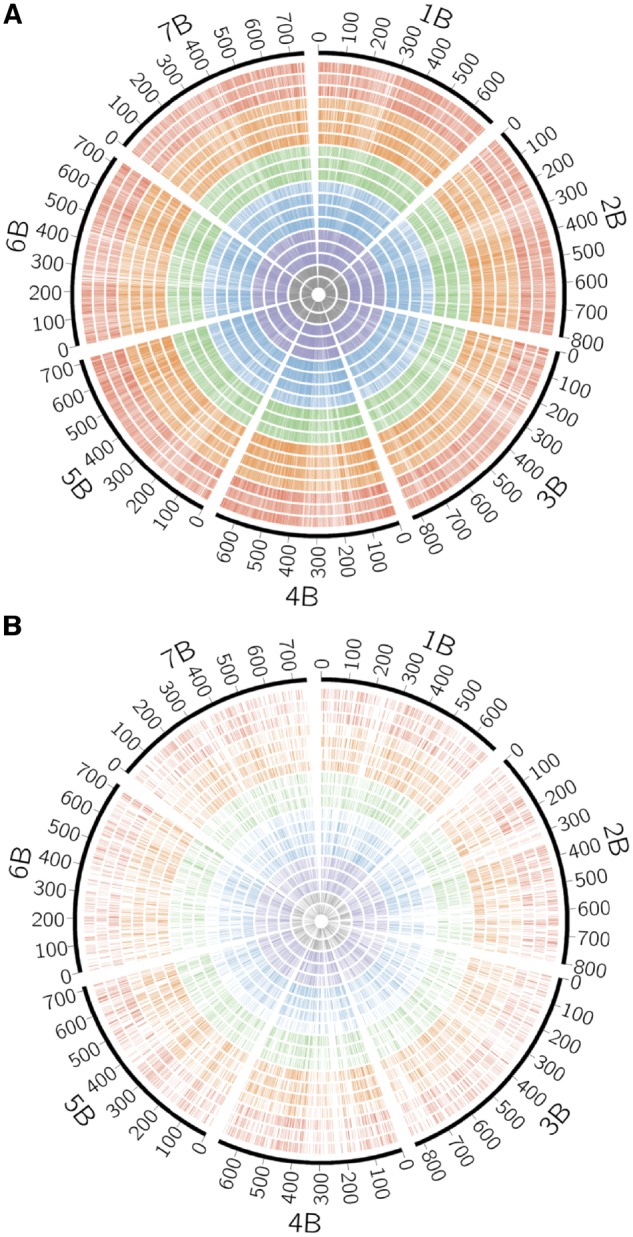
Distribution of SNPs (A) and indels (B) between the *T. aestivum* cv. Chinese Spring (CS) B genome and each of the 19 accessions of section Sitopsis species on the physical map of the CS B genome. From outer to inner circle, CS B genome (scale in Mb) and chromosome number (black), *Ae. speltoides* ssp. *ligustica* KU-2236, KU-7716, and KU-7848 (red); *Ae. spletoides* ssp. *speltoides* KU-2208A, KU-14601, KU-14605, and KU-12963a (orange); *Ae. longissima* KU-5752, KU-14624, and KU-14635 (green); *Ae. searsii* KU-5755, KU-6142, KU-6143, and KU-14651 (blue); *Ae. sharonensis* KU-14661, KU-14663, and KU-14668 (purple); and *Ae. bicornis* KU-5784 and KU-14613 (grey).

To confirm that RNA sequencing could identify genome-wide SNPs and indels, the chromosomal distribution of SNPs and indels was examined (Fig. 2). SNPs and indels identified in all of the tested accessions of Sitopsis species and the B genome entirely covered all of the chromosomes, with no clear difference in the distribution of SNPs and indels among Sitopsis species. Regions with scant or abundant SNPs on the chromosomes were quite consistent between species. For each chromosome, the number of SNPs ranged from 4,194 to 21,175, and the number of indels ranged from 34 to 317, with the high variance reflecting differences in SNP and indels numbers within species but not between species (Supplementary Fig. S3).

### 3.2. Phylogenetic relationship between Sitopsis species and B genome of bread wheat

To clarify the phylogenetic relationship and nucleotide divergence between species, we estimated sets of non-redundant SNPs anchored to each chromosome of the B genome in the 19 tested accessions of Sitopsis species and the B genome of Chinese Spring, with/without three outgroup species: *T. urartu*, *Ae. umbellulata*, and *Ae. tauschii*. Without the outgroup species, 30,589 non-redundant SNPs were obtained. When the outgroup species were included, 39,148 non-redundant SNPs were identified. These sets of non-redundant SNPs covered all of the chromosome of the B genome (Supplementary Fig. S4), allowing evolutionary analyses of section Sitopsis based on genome-wide polymorphisms.

**Figure 3:**
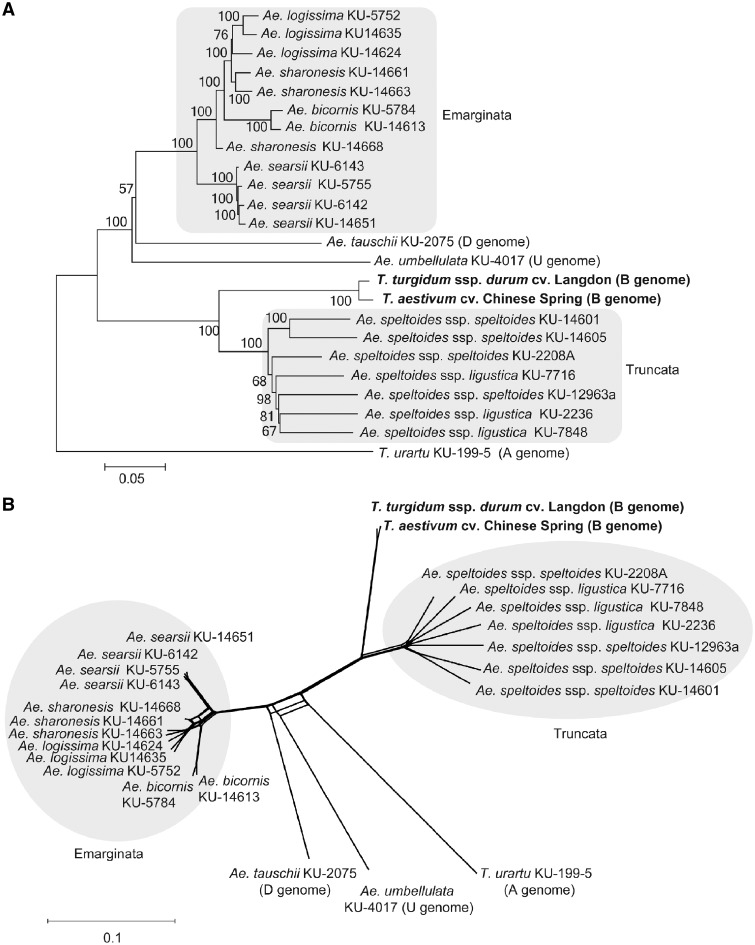
Phylogenetic relationship among the 19 accessions of section Sitopsis species (S genome), the B genomes of *T. aestivum* cv. Chinese Spring and *T. turgidum* ssp. *durum* cv. Langdon, *T. urartu* (A genome), *Ae. tauschii* (D genome), and *Ae. umbellulata* (U genome). NJ tree (A) and phylogenetic network (B) are shown. Bootstrap probabilities are shown on the branches (number of bootstrap replications = 1000). The scale bar is shown below each phylogenetic tree.

NJ and ML phylogenetic trees and a phylogenetic network tree were constructed based on the set of non-redundant SNPs with the outgroup species (Fig. 3 and Supplementary Fig. S5). The Sitopsis species were clearly divided into two clades. One clade included *Ae. speltoides* ssp. *speltoides* and *Ae. speltoides* ssp. *ligustica*, and the other clade included *Ae. longissima*, *Ae. sharonensis*, *Ae. bicornis*, and *Ae. searsii*; the two clades corresponded to subsections Truncata and Emarginata, respectively. This result was consistent with the results of previous studies based on RFLPs and AFLPs.^7^^,^^21^^,^^30^ The Emarginata clade was more closely related to *Ae. tauschii* and *Ae. umbellulata* than the Truncata clade. The B genomes of *T. aestivum* and *T. turgidum* were closely related to *Ae. speltoides* ssp. *speltoides* and *Ae. speltoides* ssp. *ligustica* in the Truncata clade. The average number of nucleotide differences and fixed nucleotide differences between *Ae. speltoides* ssp. and the B genome were the lowest in pairwise comparisons between species of section Sitopsis and the B genome (Table 3). These results supported the previous hypothesis that the B genome originated from the S genome of *Ae. speltoides*. Considering that the wheat B genome was not nested within the Truncata clade, the most recent common ancestor of *Ae. speltoides* ssp. *speltoides* and *Ae. speltoides* ssp. *ligustica* is likely the direct donor of the wheat B genome.

**Table 3:** Summary of pairwise comparisons of fixed divergence (upper) and average number of nucleotide differences (lower) between Sitopsis species

		*Ae. speltoides* ssp. *ligustica*	*Ae. speltoides* ssp. *ligustica*	*Ae. speltoides* ssp. *speltoides*	*Ae. bicornis*	*Ae. longissima*	*Ae. searsii*	*Ae. sharonensis*	B-genome	Subspecies	
										*lig*/*spel*	*lon*/*sha*
*Ae. speltoides*ssp. *ligustica*			99	9,032	8,153	8,431	7,854	6,270	0	7,693
*Ae. speltoides*ssp. *speltoides*		5,411.4		8,558	7,704	7,981	7,400	5,779	0	7,251
*Ae. bicornis*		12,785.5	12,666		1,884	3,653	1,771	13,093	7,935	1,557
*Ae. longissima*		12,412.1	12,313.5	3,239.7		2,530	121	12,175	7,110	0
*Ae. searsii*		12,052.8	11,971.9	4,318.8	3,782.8		1,976	12,498	7,356	1,841
*Ae. sharonensis*		12,070	11,965.5	3,048.5	1,720.9	3,244.9		11,811	6,821	0
B-genome		9,654	9,507.8	13,489.5	13,187	12,774.8	12,803.3		5,170	11,577
Sub-species	*lig*/*spel*	4,587.4	4,544.3	12,717.2	12,355.8	12,006.5	12,010.3	9,570.4		6,690
	*lon*/*sha*	12,241.1	12,139.5	3,144.1	1,358.7	3,513.8	1,331.2	12,995.2	12,183	

*lig/spel*: comparisons between *Ae. speltoides* ssp. *ligustica* and *Ae. speltoides* ssp. *speltoides*.

*lon*/*sha*: comparisons between *Ae. longissima* and *Ae. sharonensis*.

The two subspecies of *Ae. speltoides* were not clearly divided in the Truncata clade (Fig. 3 and Supplementary Fig. S5). The Truncata clade had longer external branches than the Emarginata clade. This observation could be explained by differences in the mating systems of the two clades: species of the Truncata clade are outcrossing, whereas species of the Emarginata clade are self-pollinating. The mating system of *Ae. speltoides* is highly outcrossing.^16^^,^^21^ RFLP analyses indicated that *Ae. speltoides* contained a higher proportion of heterozygous loci compared with other self-pollinating species of Sitopsis.^56^ Natural populations of *Ae. speltoides* would harbour nucleotide variations as heterozygous states. The tested accessions of *Ae. speltoides* had been maintained by self-pollinating for several decades in the Japanese gene bank, which could have led to fixation of one of the alleles in heterozygous sites, increasing the number of singletons within the *Ae. speltoides* accessions. This resulted in detection of a relatively large number of SNPs in the *Ae. speltoides* accessions (Fig. 1). In the Emarginata clade, *Ae. searsii* was monophyletic and separated from the other three species, whereas *Ae. bicornis* nested within *Ae. sharonensis* and *Ae. longissima*. *Aegilops longissima* diverged from *Ae. sharonensis*. The close relationship between *Ae. sharonensis* and *Ae. longissima* was consistent with previous reports^7^^,^^21^ and not inconsistent with a recent proposal that *Ae. sharonensis* is a subspecies of *Ae. longissima*.^57^

### 3.3. Identification of SNPs distinguishing subspecies ligustica/speltoides and Ae. sharonensis/Ae. longissima

**Figure 4:**
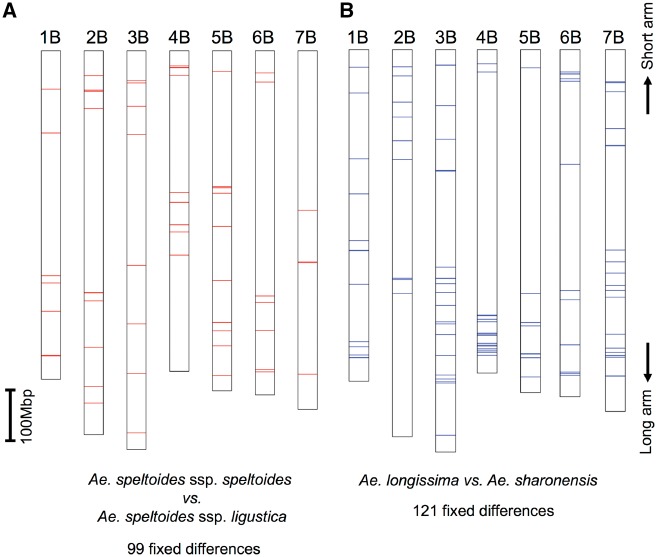
Distributions of the positions of fixed nucleotide differences between subspecies of *Aegilops speltoides* ssp. *ligustica* and *Ae. spletoides* ssp. *speltoides* (A) and between *Ae. sharonensis* and *Ae. longissima* (B) on the physical map of the B genome of *T. aestivum* cv. Chinese Spring from chromosomes 1B to 7B.

The phylogenetic tree did not discriminate well between subspecies of *Ae. speltoides* ssp. *speltoides* and *Ae. speltoides* ssp. *ligustica*, whereas the subspecies of *Ae. longissima* and *Ae. sharonensis* were distinguished. These subspecies are morphologically classified. Notably, differences in spike morphology between *Ae. speltoides* ssp. *speltoides* and *Ae. speltoides* ssp. *ligustica* can be explained by a single locus, *Lig*, located on chromosome 3S.^18^ If there are SNPs that would distinguish these subspecies (fixed nucleotide differences between subspecies), they could be related to the morphologic differences between the subspecies. Between *Ae. speltoides* ssp. *speltoides* and *Ae. speltoides* ssp. *ligustica*, 99 fixed nucleotide differences were detected (Table 3). The positions of these fixed nucleotide differences were scattered over the chromosomes (Fig. 4A). Ten of the fixed nucleotide differences caused amino acid substitutions between the two subspecies (Supplementary Table S2).

Between *Ae. longissima* and *Ae. sharonensis*, 121 fixed nucleotide differences were found (Table 3). All of the chromosomes had fixed nucleotide differences (Fig. 4B), and they were most densely located on the end of the long arm of chromosome 4B. Of all total fixed nucleotide differences, 19 caused amino acid substitutions between the two species. Genes with these fixed nucleotide differences encoded proteins involved in a variety of biological functions (Supplementary Table S3). Lateral awn elongation is a key character distinguishing *Ae. sharonensis* from *Ae. longissima*, as *Ae. longissima* lacks a lateral awn.^22^ The heading time and growth habitats of these two closely related species are also distinct.^22^ The distal region of chromosome 4S^l^, in which many fixed nucleotide differences accumulated, might control the morphologic and physiologic differences between *Ae. sharonensis* and *Ae. longissima*. Identification of the causal genes is a focus for future research.

### 3.4. Contrasting pattern of nucleotide divergence in the distal and proximal regions of the chromosomes

**Figure 5:**
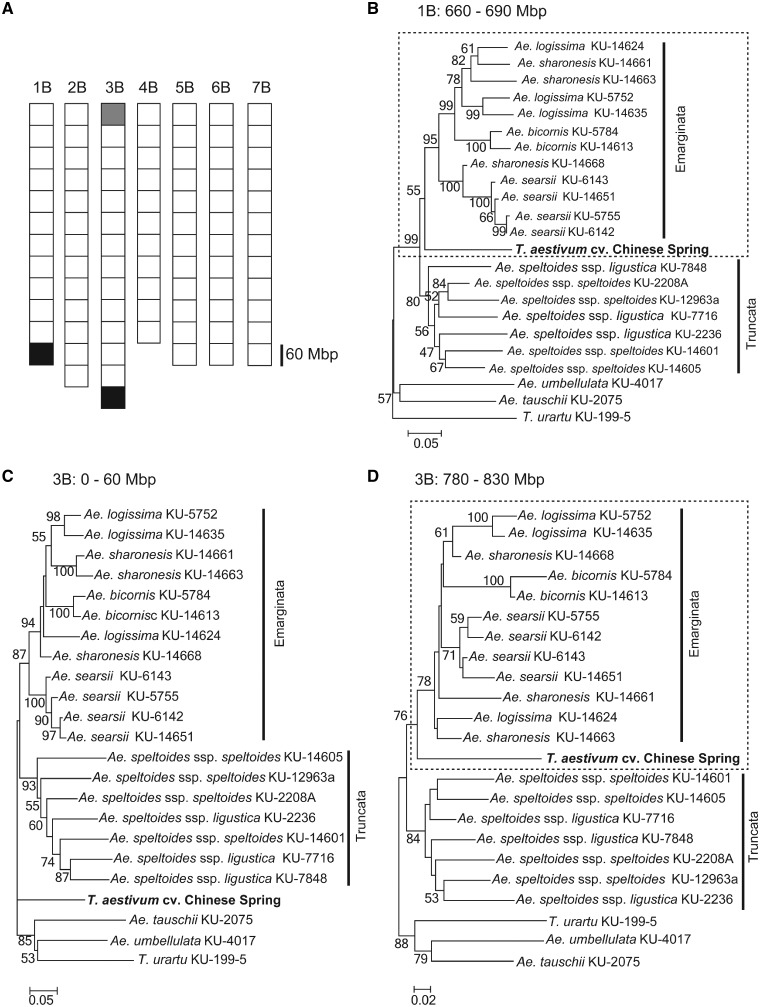
Irregular topologies of the phylogenetic trees in the distal chromosomal regions. The chromosomal regions were divided into 86 segments of 60 Mbp each. NJ trees were constructed based on non-redundant SNPs located in each segment. Squares on the chromosomes in panel (A) correspond to the 86 segments. Phylogenetic trees of the white squares showed that the B genome of *T. aestivum* cv. Chinese Spring was the most closely related to *Ae. speltoides* in the section Sitopsis. This observation was consistent with those for trees constructed based on all non-redudnant SNPs (Fig. 5). Phylogenetic trees of the black squares showed that the B genome was closely related to Emarginata clades. A phylogenetic tree of the grey square showed that the B genome was located outside of Sitopsis species. Trees with irregular topologies at the end of the long arm of chromosome 1B (B) and the short arm (C) and long arm (D) of chromosome 3B are shown. Bootstrap probabilities with over 50% and scale bars are shown for each tree.

Some previous studies suggested the possibility of introgression from several parental Sitopsis species, supporting a polyphyletic origin of the wheat B genome.^33–35^ If introgression contributed to the origin of the wheat B genome, the phylogenetic relationship between species could possibly be verified based on chromosomal positions. To test this hypothesis, NJ trees were constructed for 60-Mbp regions on each chromosome (Fig. 5). Of a total of 86 phylogenetic trees, 83 exhibited a similar topology to that of trees based on entire chromosomes, in which the B genome of Chinese Spring was closely related to the Truncata clade including *Ae. speltoides* (Fig. 3). Two trees indicated that the B genome was closely related to the Emarginata clade (Fig. 5). These irregular trees were detected at the end of the long arm of chromosomes 1B and 3B. In the other tree, the B genome at the end of the short arm of chromosome 3B was located outside the Sitopsis species.

**Figure 6:**
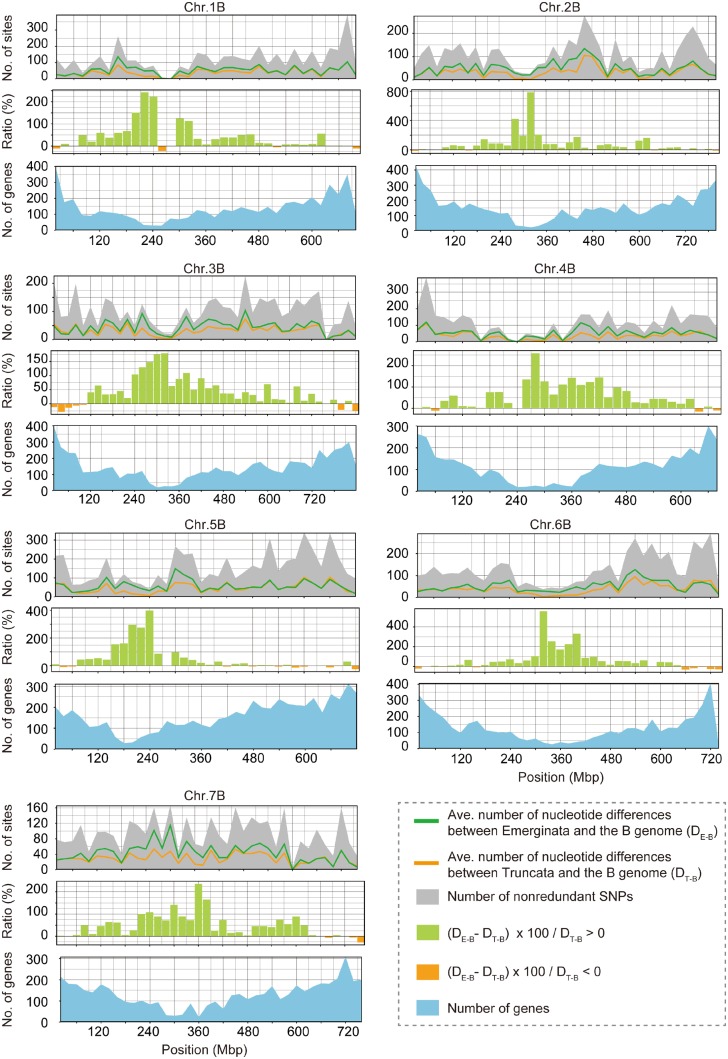
Contrasting pattern of nucleotide divergence in the distal and proximal regions of B-genome chromosomes. Average number of nucleotide differences per 20 Mbp between species in Truncata (=*Ae. speltoides* spp.) and the B genome of *T. aestivum* cv. Chinese Spring (D_T-B_) and between Emarginata species and the B genome (D_E-B_) are plotted on each chromosome. The distributions of these differences are shown by line graphs (orange and green) in the top panels. Area charts in grey colour in the top panels denote the distribution of the total number of non-redundant SNPs per 20 Mbp in Sitopsis species and the B genome. Middle panels show the distribution of the ratio expressing disparity between the two genetic divergences ([D_E-B_ − D_T-B_] × 100/D_T-B_) along each chromosome using bar charts. Area charts in blue colour indicate the distribution of the number of genes along each chromosome.

We estimated the average number of nucleotide differences between the B genome of Chinese Spring and each of the Truncata and Emarginata clades as a parameter of genetic divergence between the B genome and each clade (Fig. 6). In the distal regions of the chromosomes, genetic divergence between the B genome and Emarginata clade was slightly less than that between the B genome and Truncata clade, whereas in the proximal regions of the chromosomes, genetic divergence between the B genome and Emarginata clade was greater than that between the B genome and Truncata clade. The proximal chromosomal regions tended to exhibit conspicuous disparity in terms of genetic divergence ([D_E-B_ − D_T-B_]/D_T-B_ × 100 > 0 in Fig. 6), but the range of this disparity differed among the chromosomes. Almost the entire region (∼660 Mbp) of chromosome 3B exhibited clear disparity. In contrast, on chromosome 5B, the region exhibiting disparity was limited to within about 360 Mbp of the long arm. Except for chromosome 7B, the disparity appeared to be negatively correlated with the total number of non-redundant SNPs in Sitopsis species and the B genome. In chromosomal regions with larger number of non-redundant SNPs, genetic divergence between the B genome and Emarginata clade increased to the same extent as that between the B genome and Truncata clade. In addition, the disparity also reflected the number of genes; regions with fewer genes were found to exhibit greater disparity. Interestingly, this contrasting pattern of genetic divergence between the proximal and distal chromosomal regions corresponded to the gradient recombination rate along the chromosomes.^58^

In common wheat, the recombination rate and chromosomal gene density increase as the centromeric region recedes.^58^^,^^59^ Multiple genes created by gene duplication are more frequently located in the distal regions of the chromosomes, where they potentially drive increases in gene density and the recombination rate.^55^ This observation coincides with the observed positive correlation between the disparity of genetic divergence and number of genes (Fig. 6). Incomplete lineage sorting (ILS) is known to generate gene trees in which the topology is discordant with that of species trees and tends to more frequently occur in rapid successions of speciation events.^60^^,^^61^ If speciation events in section Sitopsis had occurred over a relatively short time, ILS in the ancestral population of Sitopsis represents a potential factor blurring the phylogenetic relationship between Sitopsis species and the B genome. ILS is positively correlated with recombination.^61^^,^^62^ Indeed, distal chromosomal regions with an irregular phylogenetic topology (Fig. 5) exhibited more complex reticulate structures in the network tree compared with whole-chromosomal regions (Fig. 3B and Supplementary Fig. S6). In the interspecies comparisons, the internal reticulate structures represent data conflicts caused by phenomena such as ILS.^63^ Therefore, ILS could explain the irregular topology of phylogenetic trees in Sitopsis species (Fig. 5) and the unclear disparity of genetic divergence (Fig. 6) that were prominent in the distal chromosomal regions with a higher recombination rate.

In the present study, a large number of SNPs and indels were discovered in the wheat B genome and five Sitopsis species based on RNA sequencing of leaf-derived transcripts. The polymorphic data would be useful for developing genome-wide markers on the S-genome chromosomes as performed for the wild diploid relatives, *Ae. tauschii* and *Ae. umbellulata*.^38^^,^^39^

In conclusion, the present phylogenetic analyses based on genome-wide polymorphisms suggest that the B genome of common wheat was derived from the S genome of *Ae. speltoides*. A few chromosomal regions demonstrated the clearly exceptional relationship between the B genome and Sitopsis species, whereas the irregular topology observed could be explained by higher recombination rates in the distal regions of wheat chromosomes. Therefore, based on genome-wide polymorphisms identified from the RNA sequencing data, all of the chromosomal regions of the wheat B genome could have originated from the S genome of *Ae. speltoides*. Moreover, the failure of pairing between homoeologous chromosomes between the B and S genomes during meiosis could be due to factors associated with highly evolved regions or intergenic regions. The alignment rate to B genome of *Ae. speltoides* was not as high as those of *Ae. bicornis* and *Ae. searsii* (Table 2). Of the transcripts that were entirely covered with unaligned RNA sequencing reads, 18% encoded F-box proteins and disease resistance proteins such as NBS-LRR (Supplementary data S1). Positive selection is known to act on these protein genes and increase nucleotide substitutions between species.^64–66^ After separation of the B genome from the S genome, the different selective pressure between B and S genomes may contribute to enhancing their genetic differentiation. In addition, distinct patterns of accumulation of repetitive sequences could have led to the differential distribution of heterochromatic regions between the B and S genomes. To elucidate the molecular nature of the differentiation of the B and S genomes, future studies should compare the repetitive sequences over all chromosomal regions.

## Data availability

All read sequences were deposited into the DDBJ/GenBank/MMBL database with accession number DRA007097. RNA sequencing data for the outgroup species were used in the DDBJ/GenBank/MMBL database: BioProject PRJDB4683 for *Ae. tauschii* KU-2075 and DRA006404 for *Ae. umbellulata* KU-4017.

## Supplementary Material

Supplementary Data
