# Supplementary material for: Origin of wheat B-genome chromosomes inferred from RNA sequencing analysis of leaf transcripts from section Sitopsis species of Aegilops
Source: DNA Res. 2019 Jan 30;26(2):171–82. doi: 10.1093/dnares/dsy047 (PMC6476730; doi:10.1093/dnares/dsy047)
Supplement: Supplementary Data [file dsy047_supp.zip › dsy047-Suppl_data/dsy047_SuppleFigs.pdf]

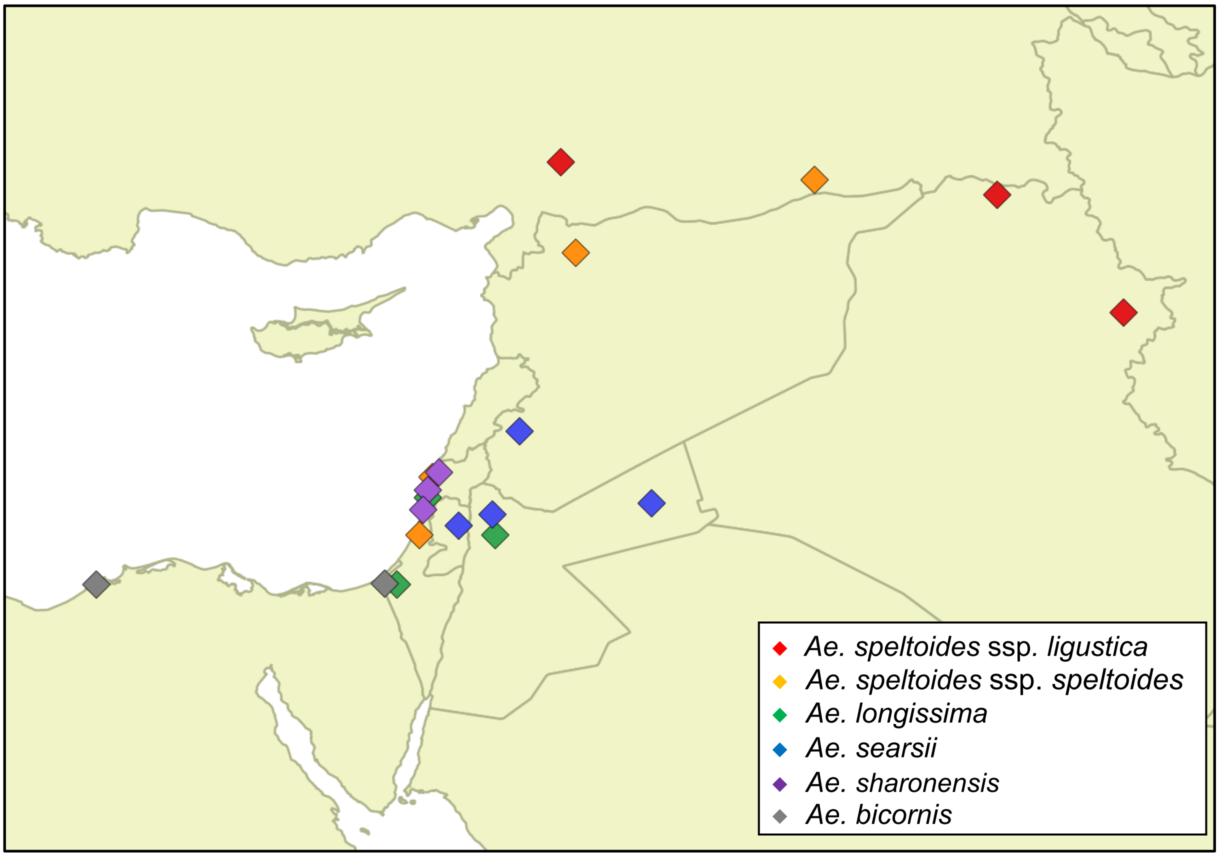

Supplementary Fig. 1. Geographic distribution of the origin of 19 accessions in section Sitopsis species shown on a map of the northwestern part of the Middle East.

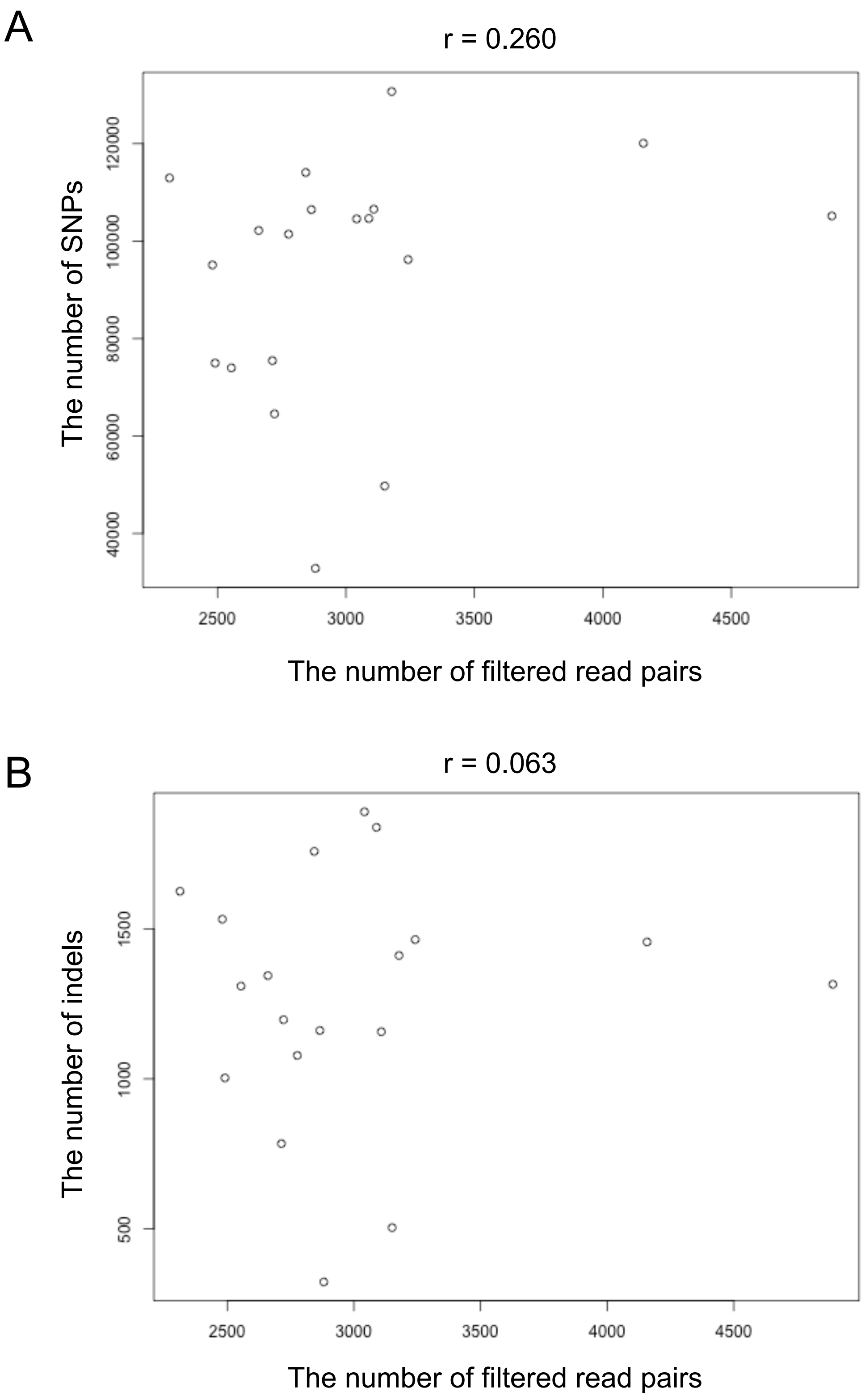

Supplementary Fig. 2 Dot plots of the number of SNPs and filtered read pairs (A) and number of indels and filtered read pairs (B). Pearson correlation (r) was calculated for each plot and indicated no significant correlation (A: p-value = 0.28 and B: p-value = 0.80).

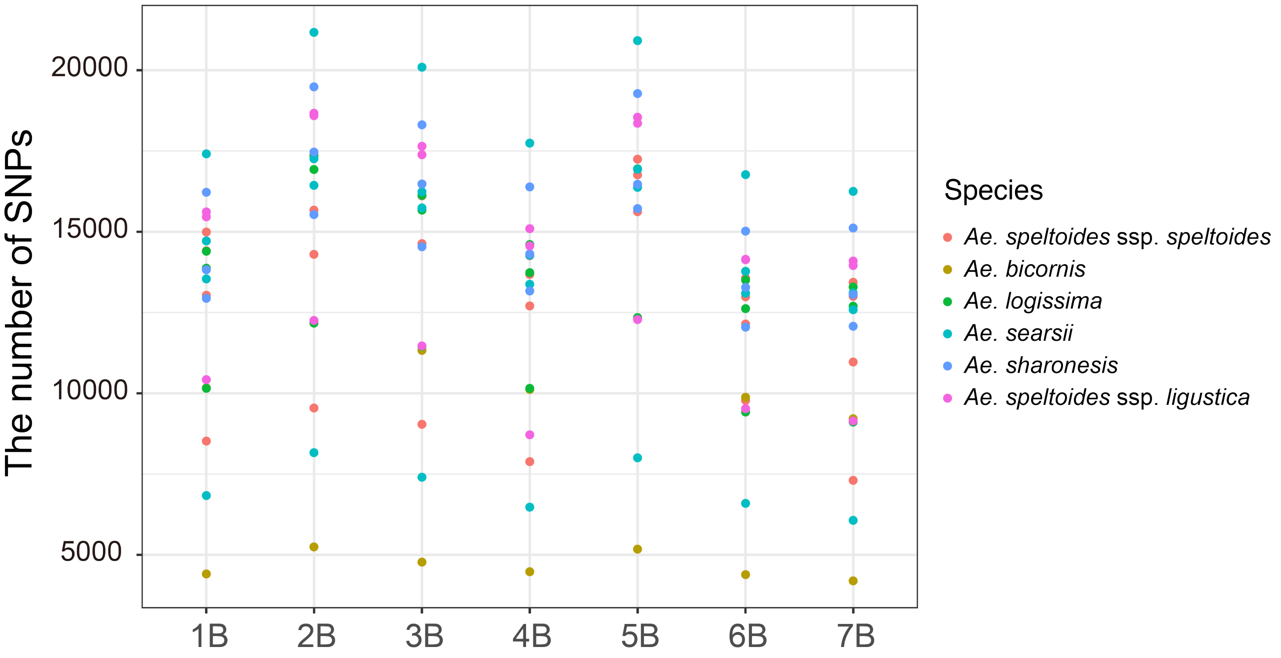

Supplementary Fig. 3 Dot plot of the number of SNPs between the B genome of *T. aestivum* cv. Chinese Spring and each of the 19 accessions of section Sitopsis species on each chromosome.

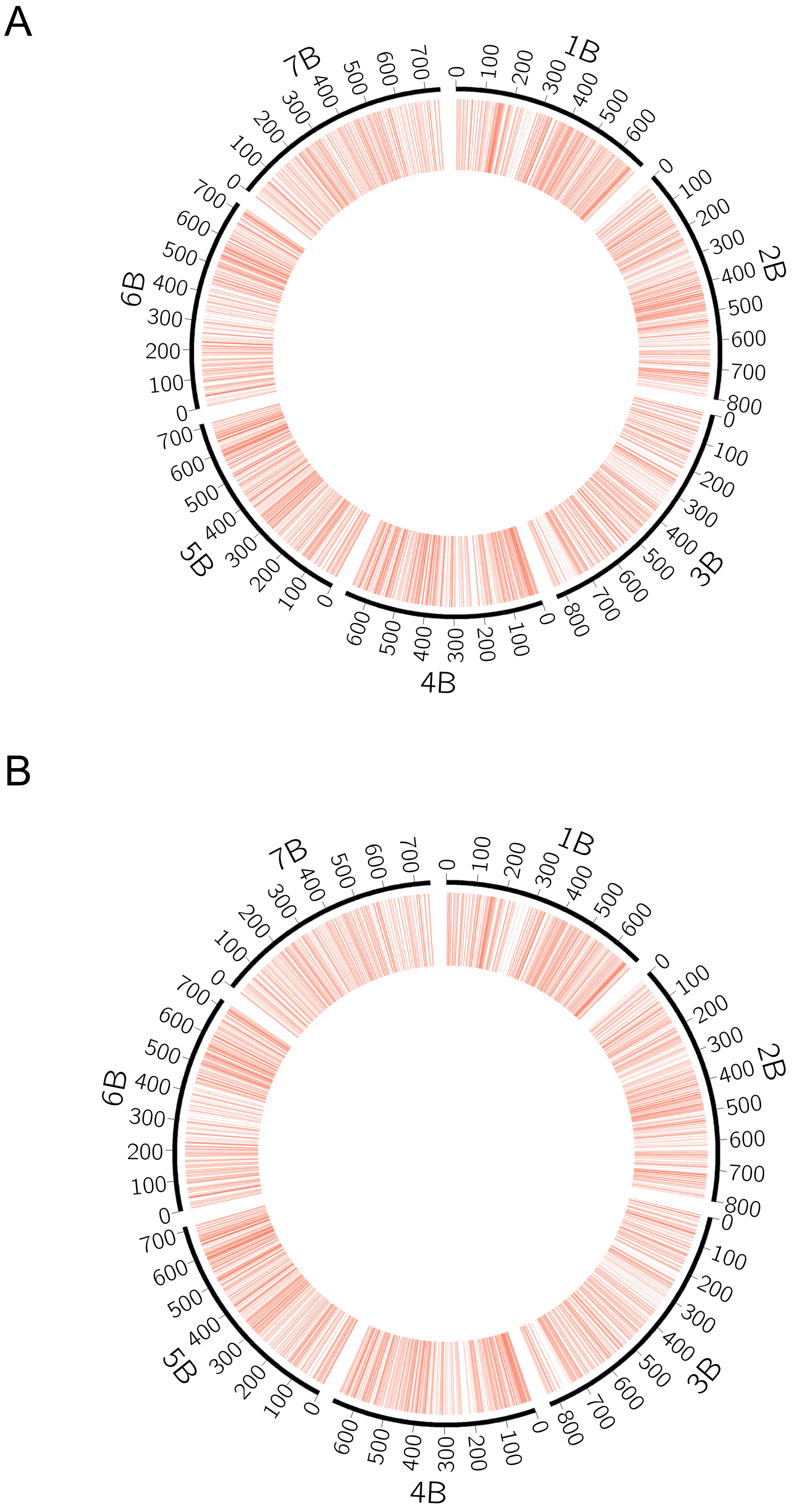

Supplementary Fig. 4 Distribution of nonredundant SNPs with outgroups (A) and without outgroups (B) in the 19 accessions of section Sitopsis species and the B genome of *T. aestivum* cv. Chinese Spring on the physical map of the B genome.

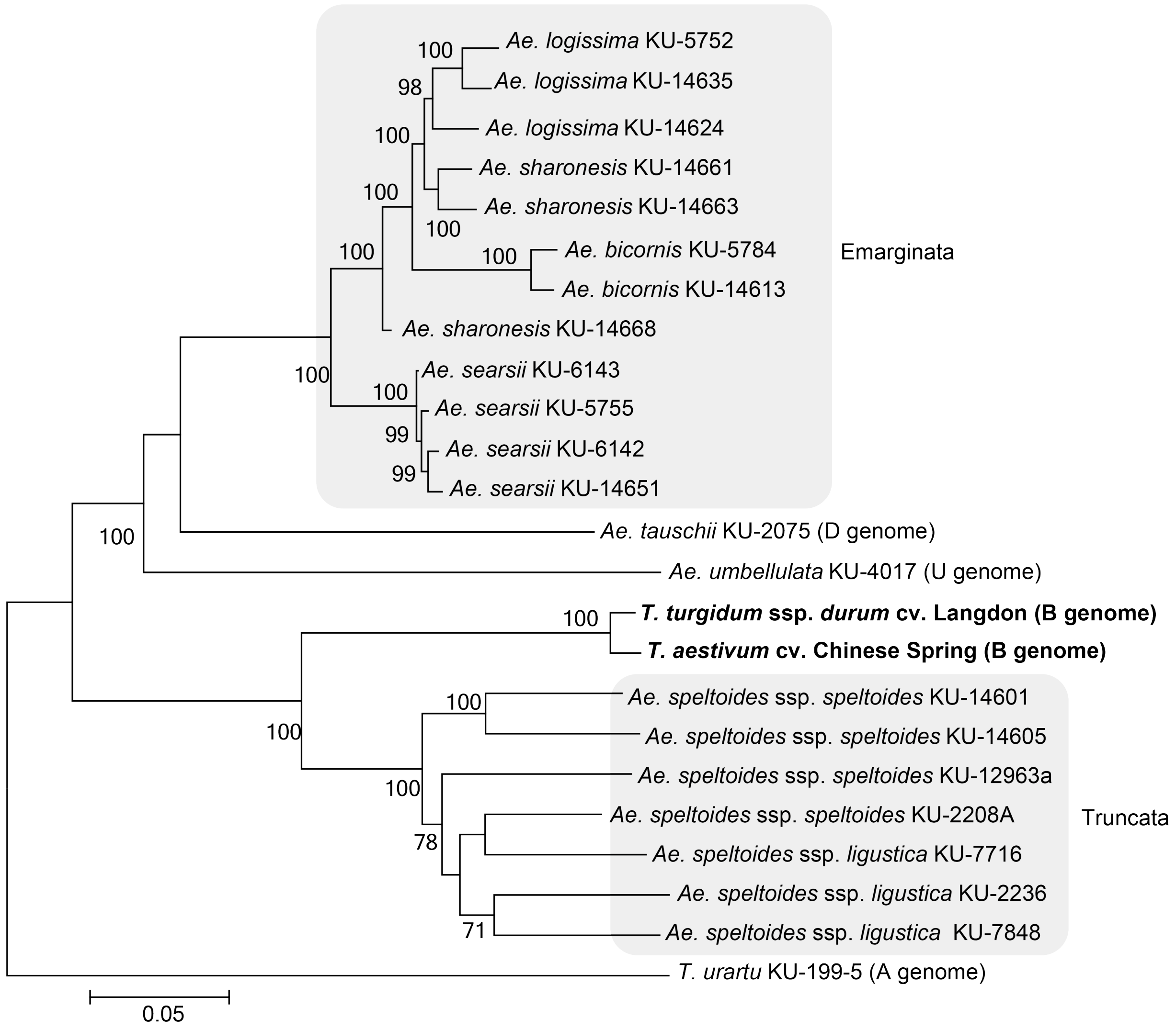

Supplementary Fig. 5 Phylogenetic relationship among the 19 accessions of section Sitopsis species (S genome), the B genomes of *T. aestivum* cv. Chinese Spring and *T. turgidum* ssp. *durum* cv. Langdon, *T. urartu* (A genome), *Ae. tauschii* (D genome), and *Ae. umbellulata* (U genome). A maximum-likelihood tree is shown. Bootstrap probabilities are shown on the branches (number of bootstrap replications = 1000). Scale bars are shown below the phylogenetic trees.

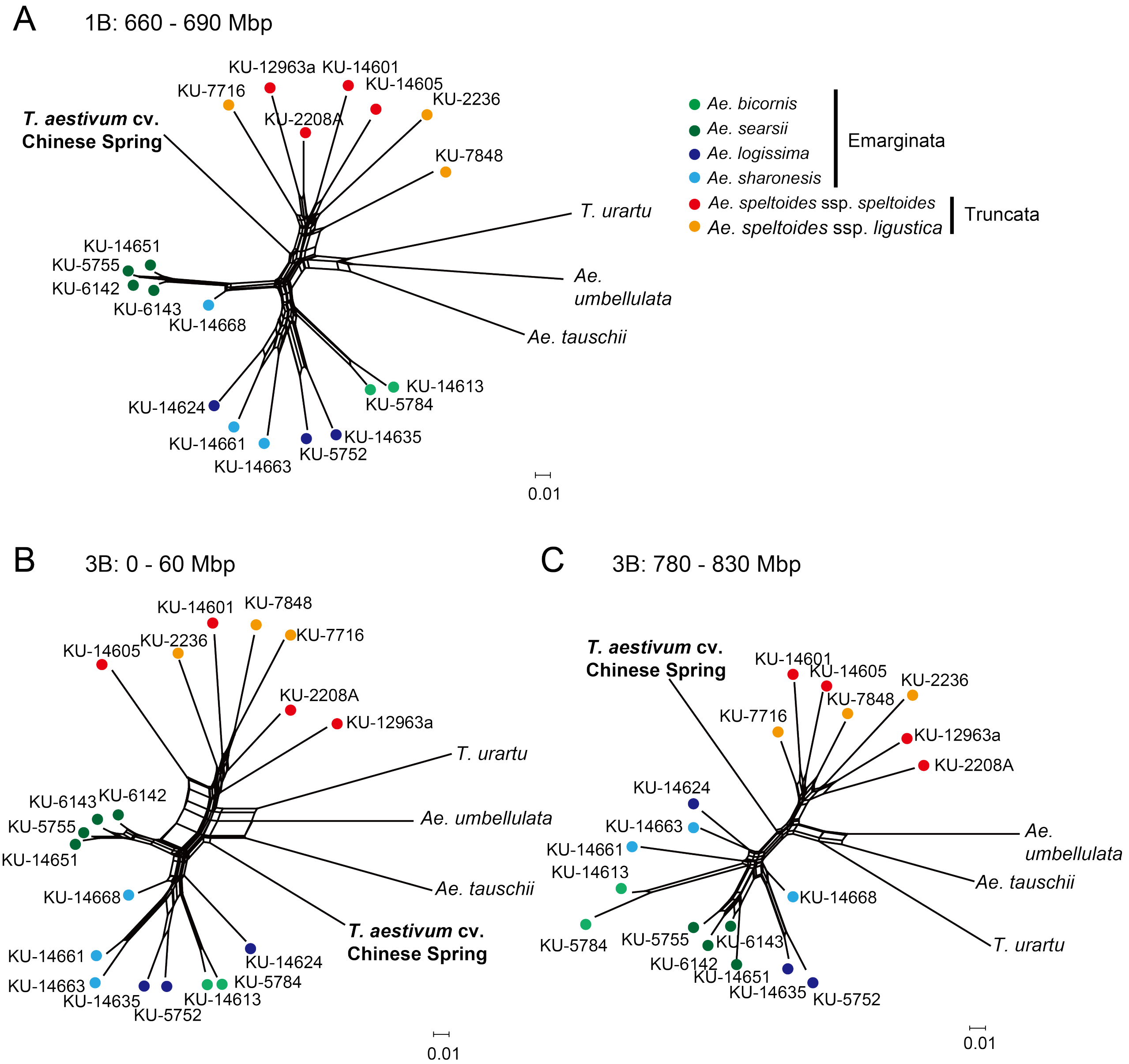

Supplementary Fig. 6. Irregular topology of phylogenetic networks in the distal chromosomal regions. Chromosomal regions were divided into 86 segments of 60 Mbp each, as shown in Figure 5. Phylogenetic trees for three segments showed an irregular topology (Figure 5). Phylogenetic network trees were constructed based on SNPs in these segments. Network trees at the end of the long arm of chromosome 1B (A) and the short arm (B) and long arm (C) of chromosome 3B are shown.
