# Supplementary material for: Origin of wheat B-genome chromosomes inferred from RNA sequencing analysis of leaf transcripts from section Sitopsis species of Aegilops
Source: DNA Res. 2019 Jan 30;26(2):171–82. doi: 10.1093/dnares/dsy047 (PMC6476730; doi:10.1093/dnares/dsy047)
Supplement: Supplementary Data [file dsy047_supp.zip › dsy047-Suppl_data/dsy047_SuppleTables.pdf]

**Supplementary Table 1** Summary of nucleotide variations per 60 Mbp on the chromosomes

| Chromosome | Positions (Mbp) | No. of genes | No. of polymorphic sites | No. of informative sites |
|---|---|---|---|---|
| 1B | 1 | 774 | 317 | 144 |
| 1B | 61 | 304 | 267 | 160 |
| 1B | 121 | 308 | 610 | 333 |
| 1B | 181 | 133 | 382 | 202 |
| 1B | 241 | 170 | 124 | 75 |
| 1B | 301 | 322 | 535 | 265 |
| 1B | 361 | 331 | 517 | 280 |
| 1B | 421 | 388 | 635 | 314 |
| 1B | 481 | 425 | 368 | 172 |
| 1B | 541 | 549 | 546 | 293 |
| 1B | 601 | 667 | 519 | 231 |
| 1B | 631 | 445 | 510 | 267 |
| 2B | 1 | 1004 | 376 | 191 |
| 2B | 61 | 523 | 414 | 209 |
| 2B | 121 | 480 | 547 | 278 |
| 2B | 181 | 380 | 453 | 211 |
| 2B | 241 | 180 | 201 | 112 |
| 2B | 301 | 106 | 246 | 147 |
| 2B | 361 | 323 | 594 | 308 |
| 2B | 421 | 362 | 818 | 448 |
| 2B | 481 | 412 | 529 | 244 |
| 2B | 541 | 417 | 260 | 131 |
| 2B | 601 | 464 | 312 | 136 |
| 2B | 661 | 610 | 445 | 227 |
| 2B | 741 | 1041 | 642 | 330 |
| 3B | 1 | 909 | 424 | 215 |
| 3B | 61 | 459 | 413 | 204 |
| 3B | 121 | 400 | 446 | 229 |
| 3B | 181 | 286 | 248 | 139 |
| 3B | 241 | 188 | 367 | 191 |
| 3B | 301 | 88 | 162 | 91 |
| 3B | 361 | 288 | 483 | 220 |
| 3B | 421 | 375 | 585 | 303 |
| 3B | 481 | 341 | 553 | 275 |
| 3B | 541 | 482 | 442 | 206 |
| 3B | 601 | 412 | 427 | 208 |
| 3B | 661 | 486 | 499 | 239 |
| 3B | 721 | 705 | 221 | 123 |
| 3B | 771 | 710 | 235 | 112 |

**Supplementary Table 1** (Continued)

| Chromosome | Positions (Mbp) | No. of genes | No. of polymorphic sites | No. of informative sites |
|---|---|---|---|---|
| 4B | 1 | 670 | 871 | 404 |
| 4B | 61 | 419 | 560 | 287 |
| 4B | 121 | 275 | 312 | 164 |
| 4B | 181 | 146 | 171 | 79 |
| 4B | 241 | 67 | 209 | 90 |
| 4B | 301 | 87 | 350 | 175 |
| 4B | 361 | 286 | 558 | 283 |
| 4B | 421 | 340 | 594 | 290 |
| 4B | 481 | 382 | 360 | 186 |
| 4B | 541 | 422 | 598 | 268 |
| 4B | 611 | 901 | 656 | 330 |
| 5B | 1 | 548 | 542 | 296 |
| 5B | 61 | 369 | 353 | 167 |
| 5B | 121 | 216 | 559 | 319 |
| 5B | 181 | 164 | 281 | 142 |
| 5B | 241 | 330 | 643 | 362 |
| 5B | 301 | 368 | 754 | 387 |
| 5B | 361 | 403 | 478 | 232 |
| 5B | 421 | 574 | 473 | 247 |
| 5B | 481 | 632 | 609 | 300 |
| 5B | 541 | 632 | 768 | 366 |
| 5B | 601 | 682 | 862 | 414 |
| 5B | 651 | 816 | 480 | 227 |
| 6B | 1 | 831 | 404 | 204 |
| 6B | 61 | 418 | 402 | 211 |
| 6B | 121 | 443 | 398 | 214 |
| 6B | 181 | 309 | 605 | 333 |
| 6B | 241 | 178 | 236 | 113 |
| 6B | 301 | 102 | 175 | 90 |
| 6B | 361 | 122 | 312 | 163 |
| 6B | 421 | 265 | 521 | 261 |
| 6B | 481 | 347 | 691 | 357 |
| 6B | 541 | 396 | 808 | 442 |
| 6B | 601 | 440 | 423 | 242 |
| 6B | 661 | 888 | 931 | 429 |

**Supplementary Table 1** (Continued)

| Chromosome | Positions (Mbp) | No. of genes | No. of polymorphic sites | No. of informative sites |
|---|---|---|---|---|
| 7B | 1 | 576 | 291 | 134 |
| 7B | 61 | 468 | 340 | 157 |
| 7B | 121 | 373 | 268 | 145 |
| 7B | 181 | 275 | 477 | 246 |
| 7B | 241 | 157 | 576 | 342 |
| 7B | 301 | 146 | 284 | 171 |
| 7B | 361 | 237 | 380 | 189 |
| 7B | 421 | 366 | 437 | 188 |
| 7B | 481 | 428 | 542 | 267 |
| 7B | 541 | 411 | 253 | 131 |
| 7B | 601 | 495 | 261 | 124 |
| 7B | 681 | 1091 | 420 | 204 |

**Supplementary Table 2** List of genes with fixed nucleotide differences between *Ae. speltoides* ssp. *speltoides* and *Ae. speltoides* ssp. *ligustica*

| Chromosome | Gene ID | Description | Position[1] | Speltoides[2] | Ligustica[2] |
|---|---|---|---|---|---|
| 2B | TraesCS2B01G359300 | Peptide methionine sulfoxide reductase MsrA | 512622962 (70) | C (Ala) | A (Ser) |
| 3B | TraesCS3B01G121000 | Mini-ribonuclease 3 | 91427090 (241) | A (Ser) | G (Pro) |
| 4B | TraesCS4B01G204900 | ADP-ribosylation factor GTPase-activating protein | 436894440 (410) | A (Glu) | T (Asp) |
| 5B | TraesCS5B01G152900 | Glutaredoxin | 282046424 (243) | A (His) | G (Arg) |
| 5B | TraesCS5B01G431400 | BTB-POZ and MATH domain protein | 606024029 (324) | G (Gln) | T (His) |
| 6B | TraesCS6B01G301700 | Universal stress protein A-like protein | 540594051 (84) | T (Lys) | C (Arg) |
| 6B | TraesCS6B01G421800 | Bidirectional sugar transporter SWEET | 692429359 (179) | A (Ile) | G (Val) |
| 7B | TraesCS7B01G240200 | Sec-independent protein translocase protein TatA | 446993358 (157) | A (Asn) | G (Ser) |
| 7B | TraesCS7B01G241100 | Reticulon-like protein | 448724068 (6) | G (Glu) | C (Asp) |
| 7B | TraesCS7B01G241100 | Reticulon-like protein | 448724186 (46) | C (Pro) | T (Ser) |

[1]Position of SNPs. The position of amino acid is shown in parentheses.

[2]The nucleotide of codon is shown. The amino acid is shown in parentheses.

**Supplementary Table 3** List of genes with fixed nucleotide differences between *Ae. longissima* and *Ae. sharonensis*

| Chromosome | Gene ID | Description | Position[1] | *Ae. longissima*[2] | *Ae. sharonensis*[2] |
|---|---|---|---|---|---|
| 1B | TraesCS1B01G 148500 | ATP synthase gamma chain | 217560227 (22) | G (Arg) | C (Thr) |
| 1B | TraesCS1B01G 167100 | Eukaryotic translation initiation factor 4E | 296495517 (120) | G (Ala) | A (Thr) |
| 1B | TraesCS1B01G 287500 | Tetratricopeptide repeat (TPR)-like superfamily protein | 499592494 (382) | T (Glu) | A (Val) |
| 2B | TraesCS2B01G 151800 | NAD(P)-binding Rossmann-fold superfamily protein | 118606789 (170) | A (Met) | G (Thr) |
| 2B | TraesCS2B01G 223600 | Kinase/ putative | 213253689 (497) | G (Asp) | C (Glu) |
| 3B | TraesCS3B01G 167000 | plant/protein (Protein of unknown function/ DUF538) | 166754290 (70) | C (Arg) | T (Gln) |
| 3B | TraesCS3B01G 167000 | plant/protein (Protein of unknown function/ DUF538) | 166754291 (70) | T (Lys) | G (Gln) |
| 3B | TraesCS3B01G 331600 | Protein curvature thylakoid chloroplastic-like | 536985528 (97) | T (Met) | C (Thr) |
| 3B | TraesCS3B01G 362500 | MAR-binding filament-like protein 1-1 isoform 2 | 574265213 (202) | A (Asp) | C (Glu) |
| 4B | TraesCS4B01G 299200 | Eukaryotic translation initiation factor 3 subunit K | 585466219 (183) | G (Ala) | A (Val) |
| 4B | TraesCS4B01G 322700 | MLO-like protein | 613158429 (372) | T (Gln) | C (Arg) |
| 4B | TraesCS4B01G 355100 | Peroxisomal membrane protein 11 homolog | 646194711 (136) | T (Phe) | G (Cys) |
| 5B | TraesCS5B01G 408300 | Cold acclimation protein | 584069137 (185) | A (Thr) | C (Pro) |
| chr7B | TraesCS7B01G 044100 | Thioredoxin-like protein | 43877520 (42) | G (Val) | A (Ile) |
| 7B | TraesCS7B01G 143300 | Succinyl-diaminopimelate desuccinylase | 184195978 (253) | A (Met) | G (Val) |
| 7B | TraesCS7B01G 144100 | RING/U-box superfamily protein | 185299978 (41) | A (Leu) | G (Pro) |
| 7B | TraesCS7B01G 223000 | Adenylate kinase | 419031679 (127) | A (Lys) | C (Gln) |
| 7B | TraesCS7B01G 288500 | Beta-hexosaminidase | 524398482 (115) | A (Phe) | T (Ile) |
| 7B | TraesCS7B01G 388800 | Peptidylprolyl isomerase | 655040771 (24) | G (Ala) | C (Pro) |

[1]Position of SNPs. The position of amino acid is shown in parentheses.

[2]The nucleotide of codon is shown. The amino acid is shown in parentheses.
